# The Impact of Monkeypox Virus Infection on Pregnancy Outcome: Clinical Features and Potential Pathogenic Mechanisms

**DOI:** 10.1155/jimr/5158686

**Published:** 2026-01-18

**Authors:** Jingyi Wu, Fangbin Huang, Qingliang Zheng

**Affiliations:** ^1^ The Eighth Affiliated Hospital, Sun Yat-sen University, Shenzhen, China, sysu.edu.cn

**Keywords:** abortion, immune evasion, monkeypox virus, pathogenesis, pregnancy complications, vertical transmission

## Abstract

Monkeypox has garnered significant attention following its emergence in multiple countries and its designation as a global health emergency in 2022. There is a notable deficiency in clinical and experimental data regarding pregnancy outcomes following monkeypox infection. The complications associated with monkeypox virus (MPXV) infection, including fetal miscarriage, preterm delivery, and congenital infections, have not been adequately addressed, and there is a lack of effective clinical interventions. Two clades of MPXV have been identified: clades I and II, with clade I being the predominant strain previously associated with adverse pregnancy outcomes. Clade IIb has also been implicated in potential vertical transmission from mother to child. Infection of a pregnant individual with MPXV can lead to fetal conditions, such as generalized diffuse herpes hydatidis and extensive hemorrhage within the placenta. MPXV is thought to evade the immune response primarily by inhibiting interferon (IFN)‐mediated antiviral effects, suppressing host immune recognition, dampening host inflammatory responses, and modulating cellular apoptosis. This review aims to provide a comprehensive overview of the clinical manifestations, potential routes of vertical transmission, and possible pathogenic mechanisms of MPXV infection in pregnant women, thereby serving as a valuable reference for future diagnostic, therapeutic, and pharmacological strategies in managing MPXV‐related pregnancy complications.

## 1. Introduction

Mpox is a zoonotic disease caused by the monkeypox virus (MPXV). The first reported human case was in a 9‐month‐old infant in Congo in 1970 [1]. In May 2022, multiple cases of mpox were detected in several nonendemic countries, and on July 23 of the same year, the World Health Organization (WHO) declared mpox a global health emergency. From January 2022 to January 2023, more than 84,000 cases of mpox were detected in more than 112 countries/areas [2]. MPXV can be transmitted from person to person through respiratory droplets, close contact, and direct contact with skin lesions or contaminants from infected persons [3–5]. Despite tens of thousands of global mpox cases, systematic studies on infection during pregnancy are severely lacking—a critical knowledge gap highlighted by the current outbreak. Existing evidence, largely from small‐scale clinical observations and case reports, already points to significant risks. For example, an investigation of the 2022–2024 clade I outbreak in the Democratic Republic of the Congo (DRC) reported severe pregnancy outcomes, including miscarriage or stillbirth, in 50% (4 of 8) of confirmed pregnant women [6]. Concurrently, case reports on clade II—the strain driving the ongoing global outbreak—describe spontaneous abortion, stillbirth, and intrauterine vertical transmission. Large‐scale systematic analyses further indicate that clade IIb is the predominant clade linked to novel transmission routes, including mother‐to‐child transmission, in the current outbreak. Together, evidence from diverse geographic regions and viral lineages demonstrates that MPXV threatens both pregnant individuals and their fetuses, carrying a tangible risk of vertical transmission. Nevertheless, robust clinical evidence specifically for clade IIb in pregnancy remains scarce, highlighting a critical knowledge gap that demands urgent investigation through multinational cohort studies. Consequently, there is limited data on the effects of MPXV infection during pregnancy on the fetus and placenta and the ability to transmit vertically [7]. Although there are fewer studies on MPXV infection during pregnancy, cases and studies, have concluded that while most cases do not result in maternal death, adverse pregnancy outcomes such as fetal miscarriage may occur [8]. A clinical case demonstrated that MPXV‐infected pregnant women were capable of transmitting the virus to their offspring during the perinatal period, and the stillborn fetus had clinical signs of diffuse hydatidiform herpes on the head and body, marked hepatosplenomegaly with abdominal effusion, and severe liver damage. The placenta exhibits pathological findings—specifically, massive punctate and diffuse hemorrhage—that are analogous to the cutaneous manifestations seen in MPXV‐infected patients [9].

MPXV comprises two distinct clades with significant pathobiological differences: clade I (Central African Basin clade) and clade II (West African clade) [10]. Clade I has historically been associated with higher virulence and is primarily linked to zoonotic transmission and spread through close contact in endemic areas [11]. In contrast, clade II demonstrates lower virulence, with reported case fatality rates of less than 1% [11]. It is important to emphasize that differences in virulence between viral lineages are primarily observed in animal model studies. This is because the epidemiological context of outbreaks, accessibility to medical resources, and fundamental characteristics of affected populations vary significantly—factors that make direct comparisons challenging. In human epidemics, such differences are less readily discernible [12]. However, this framework has been disrupted by recent evolution. Since 2022, clade IIb—a descendant of clade II—has triggered an unprecedented global outbreak, marked by a distinct epidemiological shift toward efficient transmission via sexual contact networks, particularly among men who have sex with men (MSM) [13, 14]. Concurrently, the emergence of clade Ib in regions including the DRC has also demonstrated enhanced human‐to‐human transmission through sexual contact, raising new public health concerns [15]. These fundamental differences in virulence, affected populations, and transmission modes between the major clades suggest the potential for markedly divergent impacts on pregnancy outcomes and vertical transmission risk—a critical area in which systematic research remains notably lacking.

The global mpox epidemic, initiated by clade IIb in 2022 and continuing to the present, coupled with emerging evidence of severe outcomes in pregnant women, has rendered this a critical area of research. However, systematic comparisons of MPXV clinical course and severity between pregnant and nonpregnant individuals are still lacking, leaving the unique impact of pregnancy incompletely defined. This article reviews the clinical features, pregnancy outcomes, possible routes of vertical transmission from mother to child, and the pathogenic mechanisms of MPXV infection, with an enhanced focus on integrating available comparative data to assess pregnancy‐specific risks.

## 2. MPXV and Its Clinical Features After Infection

### 2.1. Overview of MPXV

**Figure 1:**
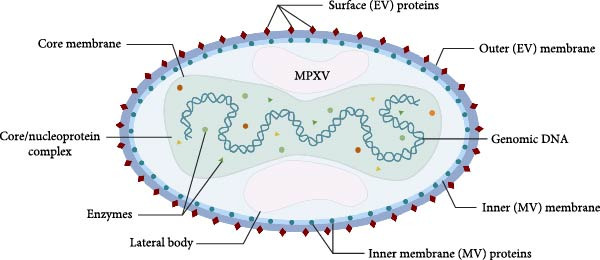
Structural characterization of MPXV. MPXV consists of five main components: a biconcave dumbbell‐shaped core, lipoprotein membrane, lateral body, surface tubules, and genomic DNA.

Mpox is caused by the MPXV, an enveloped double‐stranded DNA (dsDNA) virus of the Orthopoxvirus genus in the Poxviridae family [16]. The MPXV genome is ~197.2 kb in length and contains ~223 open reading frames (ORFs). The genome can be divided into three regions: at both ends of the genome are inverted terminal repeats (ITRs) of 10 kb in length; the core region in the middle is responsible for transcription, replication, and assembly of viral particles, and encoding ~181 proteins [3] (Figure 1). MPXV is divided into clade I (formerly known as the “Congo Basin clade” or “Central Africa clade,” which is subdivided into Ia and Ib) and clade II (formerly known as the “West Africa clade,” which is now subdivided into IIa and IIb). Based on a systematic review of available clinical reports, the reported case fatality rate for clade I was 10.6%, which is numerically higher than the 3.6% observed for clade II [11]. However, it is important to note that these estimates are derived from heterogeneous clinical cohorts and epidemiological contexts, and findings from animal models provide more controlled evidence for the difference in virulence [12]. Although the genomic sequences between clade I MPXV and clade II differ by only 0.55%−0.56%, differences in key regions encoding important virulence genes cause differences in disease severity between the two clades [17].

The most significant difference between the two clades of virulence genes lies in BR‐203 (OPG015), BR‐209 (OPG016), and the immediate homologs of COP‐C3L (OPG213) [18]. BR‐203 is a direct homolog of the Mucovirus M‐T4 gene and encodes a protein that protects infected lymphocytes from apoptosis [19]. BR‐203 (OPG015) of MPXV clade I can encode a full‐length protein of 221 amino acids, whereas clade II only encodes an N‐terminal fragment of ~51 amino acids [18]. The 326 amino acid protein encoded by the BR‐209 (OPG016) gene functions as an interleukin‐1*β* (IL‐1*β*) binding protein that prevents IL‐1*β* and IL‐1 receptor binding [20]. The presence of a full‐length 326‐amino‐acid protein in clade I, reported by Chen et al. [21] and a single base insertion and a four‐base deletion near the N‐terminus in clade II resulted in two shifted mutations. The protein known as monkeypox inhibitor of complement enzymes (MOPICEs) is encoded by clade I of a direct homolog of the COP‐C3L (OPG213) gene 216 amino acids in length [22], but the gene is not present in clade II [21]. These differences in virulence genes may be partly responsible for the greater virulence of clade I than that of MPXV clade II.

### 2.2. Clinical Features Caused by MPXV Infection

Mpox is a self‐limiting disease with the main clinical manifestations of fever, rash, and enlarged lymph nodes, which usually resolve within a few weeks [23]. The incubation period of mpox ranges from 1 to 2 weeks, but it can also vary from 5 to 21 days. Most patients experience prodromal symptoms, such as fever with malaise, sweating, and fatigue, 2 days before the rash appears [24]. The rash first appears on the face and quickly spreads to the extremities, trunk, and other parts of the body in a centrifugal distribution [25], but the rash has a centripetal distribution in a few cases [26]. In early African outbreaks, rashes on the face and extremities were more common than those on the body trunk. A key clinical feature particularly relevant to obstetric practice is the high frequency of perianal and genital lesions (appearing on the penis, labia, vagina, and perianal region) reported during the 2022 outbreak (driven by Branch IIb) [27]. This shift in rash localization suggests a potential increased risk of retrograde genital tract infection and underscores the necessity for careful genital examination of pregnant women.

The rash begins with a red plaque of 2–5 mm (flat lesion at the base) and then evolves into papules (hard lesions with a slight bulge), vesicles (lesions with a slight bulge), pustules (lesions filled with yellow fluid), and scabs, which subsequently slough off and leave a hyperpigmented scar [28, 29]. Vesicles and pustules are mostly spherical, ranging from 0.5 to 2 cm in diameter, with a hard texture, clear boundaries, and deep involvement. The central depression is umbilical fossal‐like and may be accompanied by obvious itching and pain [29]. Usually, the appearance of a rash marks the beginning of the infectious phase, and MPXV is contagious until the crusts fall off. The development of the rash is slow, and the lesions in the same site are often in the same stage of development. Each stage lasts 1–2 days; pustular period can last 5–7 days. From the onset to the shedding is about 2–4 weeks; the maximum is 8 weeks [30].

Other clinical features of mpox include enlarged lymph nodes (predominantly in the neck, axilla, and inguinal areas), pharyngitis, tonsillitis, proctitis, and diarrhea. Lymph node enlargement is a distinguishing feature of MPXV infection compared to other orthopoxvirus infections [31].

## 3. MPXV Infection Leads to Pregnancy Complications

The first study in the DRC in 1980 reported that a pregnant woman infected with MPXV gave birth to a live‐born infant with a generalized rash suggestive of mpox, who died of malnutrition 6 weeks later [32].

From March 2007 to July 2011, a large‐scale study was conducted in the Sankuru Province of the DRC. 222 symptomatic patients were enrolled, of whom 81 (36%) were female patients. Four of them were pregnant and were confirmed MPXV clade I infection [9]. One woman with mild disease delivered a newborn at full term without clinical features of monkeypox infection. Three cases of moderate‐to‐severe maternal infection had adverse pregnancy outcomes: two spontaneous early pregnancy abortions at 6 weeks of gestation and one mid‐gestational loss of a fetus at 18 weeks of gestation. One of the stillborn fetuses from a moderately infected MPXV mother consisted of diffuse cutaneous maculopapular lesions involving the skin of the head, the trunk (abdomen, back and chest), and the extremities (palms and soles of the hands and feet). In addition, Hydrops fetalis was detected, and there was marked hepatomegaly with peritoneal effusion. Massive punctate diffuse hemorrhage of the placenta with high viral loads (> 10^7^ genome copies/mL) detected in fetal tissue, umbilical cord, and placenta [9, 33]. This strikingly high fetal loss rate (75%) appears to differ markedly from the low mortality and minimal risk of severe sequelae reported for nonpregnant individuals with MPXV in endemic settings [11], suggesting that pregnancy may be a critical determinant of disease severity.

Mpox cases increased globally during 2022–2023 [23], which was mainly caused by clade II [33]. More than 112 countries have reported MPXV infection, including 87,970 confirmed cases and 140 deaths [28], including 58 in pregnant women. Because the disease caused by clade II is mildly symptomatic, with an overall mortality rate of 0.1%–3.6%, and because only a small percentage of pregnant women are infected, there have been no reports of maternal deaths or fetal complications, although at least 58 cases of maternal infection have been reported [6, 33, 34]. In addition, four of the eight pregnant women with confirmed or suspected MPXV clade IIb infection reported by Masirika et al. had aborted fetuses, and one of the aborted fetuses had a rash similar to MPXV infection; however, none of the placentas or fetuses were autopsied [34]. This indicates that even with the less virulent clade IIb, pregnancy may still confer a heightened risk for severe fetal complications, diverging from the expected outcome in the general population. In another cohort of pregnancies described by Oakley et al., three pregnancies were infected with MPXV clade IIb during pregnancy, one of which had a spontaneous abortion at 11 weeks of gestation, and the remaining two were delivered at full term without complications [35].

MPXV clade I causes adverse pregnancy outcomes, but the severity of clade IIb causing adverse pregnancy outcomes is somewhat controversial. Thus, Nicholas P. Krabbe et al. establish a pregnant rhesus macaque model of clade IIb MPXV infection with early gestation inoculation to understand if infection results in vertical transmission and adverse pregnancy outcomes. Their observation that MPXV clade IIb in pregnant rhesus monkeys can be transmitted vertically to the fetus (MPXV was detected at the maternal–fetal interface, in amniotic fluid, and fetal tissues) and lead to adverse pregnancy outcomes similar to those associated with clade I infections, which suggests that MPXV clade IIb infections in human pregnancies also pose a threat to maternal and fetal health [36]. However, the current human data for clade IIb are too limited and fragmented to accurately define the spectrum of pregnancy risks, underscoring the imperative for large‐scale, systematic clinical studies. The key similarities and differences in clinical features and pregnancy outcomes between MPXV clade I and clade II are summarized in Table 1.

**Table 1:** Clinical features and pregnancy outcomes of MPXV infection.

Category	Clinical features/pregnancy outcomes	Clade I	Clade II	Remarks/source of evidence
General clinical features	Incubation period	1–2 weeks (5–21 days)	Similar	—
	Prodromal symptoms	Fever, malaise, sweating, fatigue (2 days before rash onset)	Similar	Lymphadenopathy is a characteristic feature of MPXV
	Rash distribution	More common on face and extremities (Centrifugal)	High frequency of genital and perianal rash	Characteristic of the 2022 outbreak
	Rash progression	Macules → Papules → Vesicles → Pustules → Crusts; Often synchronous at one site	Similar	The entire process lasts ~2–4 weeks

Pregnancy outcomes	Fetal loss rate	High (studies show 75%)	Controversial, limited case reports	—
	Typical complications	Early miscarriage, mid‐trimester fetal loss, stillbirth	Majority of reports describe no severe complications, but sporadic abortion cases exist	—
	Vertical transmission evidence	High viral load detected in fetal tissues and placenta	Limited evidence in human cases; confirmed in rhesus monkey models	Key evidence supporting the potential threat of Clade IIb

## 4. Potential Pathways of MPXV Infection From Mother to Fetus

Despite the coordinated antiviral immune responses at the maternal–fetal interface, which involve multiple cell types including trophoblasts, decidual stromal cells, and a variety of resident immune cells (such as macrophages, natural killer (NK) cells, and dendritic cells) [37], clinical case studies provide direct evidence that MPXV can be transmitted vertically: MPXV DNA was detected in the fetus and placental lesions of the infected woman; immunohistochemistry was also positive for MPXV in fetal skin and placental villous cells [33]. These findings confirm the potential for MPXV vertical transmission, although the specific molecular and cellular pathways remain incompletely elucidated. Current understanding relies heavily on inferences from structurally related orthopoxviruses, such as vaccinia virus (VACV) and cowpox virus (CPXV). The following section explores four plausible routes for vertical transmission of MPXV, as suggested by clinical case studies and analogous research. It is important to note, however, that these pathways remain hypothetical and await validation through direct investigation (Figure 2).

Figure 2Potential routes of vertical transmission of monkeypox virus to the fetus. (A) Structural features of the maternal‐fetal interface. A full‐term fetus is shown on the left, and enlarged chorionic villus bundles are shown on the right. The free chorionic villus ends are suspended in the maternal blood‐filled chorionic interstitium. Anchored chorionic villi are attached to the bottom metaphase by highly proliferating columnar cell trophoblasts. Fetal blood vessels are located inside the villi and are covered by chorionic cell trophoblasts and syncytial trophoblasts. The maternal uterine spiral artery passes through the metaphase plate and into the maternal lobe. (B) MPXV directly infects syncytial trophoblasts via trophoblast membrane fusion or megaloblast drinking action. (C) Ascending routes. MPXV can cross the cervix and reach the chorionic barrier of the placenta. The virus infects the choroid and amniotic membrane at the intercellular level to reach the amniotic fluid, which the fetus continuously inhales and swallows to become infected. (D) ① Hematogenous route. MPXV in maternal blood reaches the interchorionic blood via the uterine spiral artery, binds to syncytiotrophoblasts, and infects syncytiotrophoblasts, cellular trophoblasts, mesenchymal fibroblasts, and fetal blood. ② MPXV infection leads to the destruction of cytokines released by the innate and adaptive maternal immune response, allowing the trophoblastic epithelium to be destroyed, leading to the invasion of MPXV into the fetal bloodstream and causing fetal infection.

**Figure:**
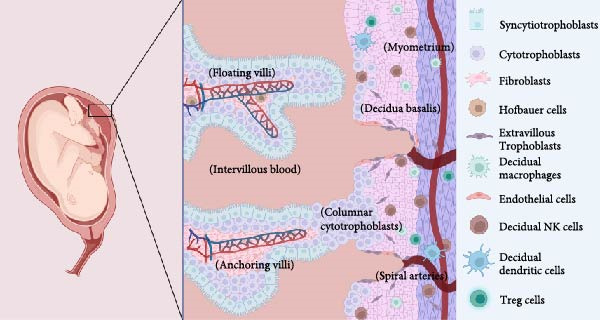
(A)

**Figure:**
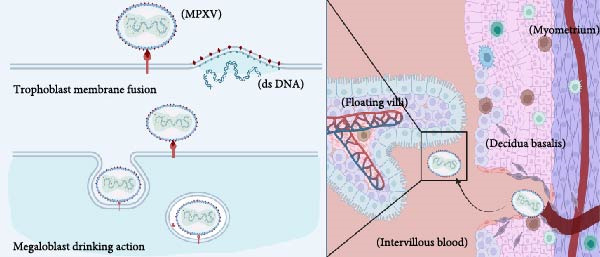
(B)

**Figure:**
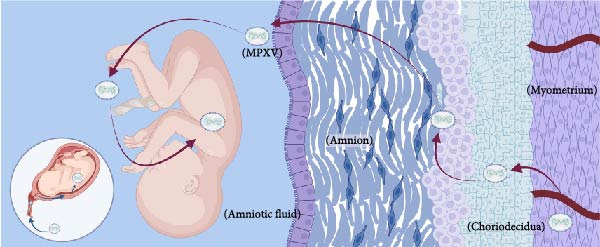
(C)

**Figure:**
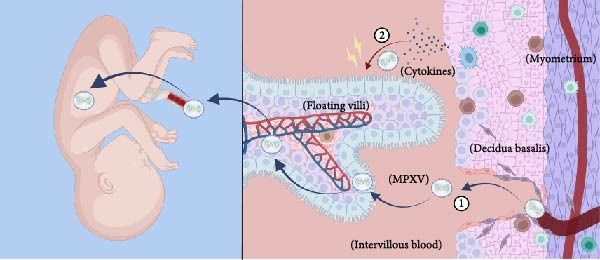
(D)

First, the ability of syncytiotrophoblasts to perform megacytosis has been demonstrated [38]. Although there is no direct evidence for MPXV, studies on the closely related VACV indicate that VACV can enter cells via micropinocytosis [39]. Given the structural and envelope similarities between MPXV and VACV, we infer that MPXV may also directly infect syncytial trophoblasts by fusion with the trophoblast membrane or by micropinocytosis [40].

Second, Krabbe et al. [36] detected a high viral DNA load in the vaginal secretions of pregnant rhesus monkeys infected with MPXV, providing some support for an ascending transmission route. Additionally, in cases of abortion caused by human cowpox (CPXV) infection, infectious virus has been detected in maternal vaginal secretions, and cytopathic effects can be observed in both the vagina and placenta 4–6 weeks after maternal infection [41]. Based on the evidence regarding orthopoxviruses, we therefore propose a hypothesis: By a comparable mechanism, MPXV may also cause genital mucocutaneous lesions (e.g., on the vulva, vagina, or cervix). The resulting local inflammation or injury can compromise the barrier function of the cervical mucus plug. This breach could enable the virus to ascend, infect the chorion and amniotic cavity, and enter the amniotic cavity. Subsequently, the fetus may become infected in utero through the continual swallowing and inhalation of contaminated amniotic fluid [42, 43].

Third, MPXV infection causes viremia, and combined with clinical evidence of high viral loads detected in the placenta and fetus of aborted fetuses [42], this strongly suggests that MPXV is likely transmitted via the bloodstream. Based on this, we propose the following model: MPXV may reach the placental chorionic interstitial space through the spiral arteries in the maternal uterus and then bind to trophoblast cells. The infection can then propagate—through intercellular junctions or by direct infection of the cytotrophoblast—deeper into the villous stroma. Ultimately, by infecting the endothelium of fetal blood vessels, the virus completes its transmission into the fetal circulation [42].

Finally, inflammation associated with MPXV infection may indirectly promote viral invasion by enabling the virus to invade the placenta or fetal blood. Studies indicate that in malaria‐infected placentas, the production of proinflammatory cytokines such as IFN‐*γ* and TNF‐*α* disrupts the integrity of the placental barrier [44]. Based on this, we hypothesize that in MPXV infection, which triggers a similar cytokine storm, the destruction of syncytiotrophoblast structure may similarly create opportunities for viral invasion into the fetal circulation. Cytokines such as IFN‐*γ* and TNF‐*α* released by NK cells; IL‐12, IL‐18, and IL‐6 released by dendritic cells; and IL‐17A released by T‐lymphocytes also can disrupt the cortical actin network on the syncytial trophectoderm [43], which contributes to the invasion of MPXV into the fetal blood. Therefore, we hypothesize that this inflammation‐mediated placental damage may serve as a contributing factor to the vertical transmission of MPXV.

## 5. Pathogenic Mechanisms of MPXV Infection

The process of MPXV infection and replication can be summarized in three stages: viral invasion; viral replication and synthesis; and viral assembly, maturation, and release [45].

### 5.1. Mechanisms of MPXV Invasion and Release

**Figure 3:**
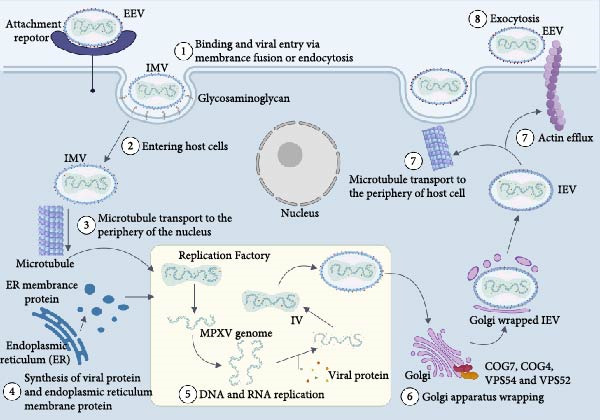
Life cycle of MPXV replication in the host cells. Both monkeypox virus IMV and EEV viral particles penetrate the host cell membrane via membrane fusion and endocytosis. MPXV particles utilize glycosaminoglycans as host receptors, and IMV enters the cytoplasm and is transported by microtubules to the perinuclear replication factories. After viral genome replication is complete, IMV is enveloped by the Golgi apparatus to form IEV, which is transported to the cell surface via actin or microtubules and released by fusion with the cell membrane to form EEV.

In the early stages of MPXV infection, two types of infectious virus particles with different structures and surface proteins are present: extracellular enveloped virus (EEV) and intracellular mature virus (IMV) (Figure 3). The IMV is enclosed by a single lipid bilayer, which is protected by a dense proteinaceous coat. This robust structure confers high resistance to environmental insults, prolonging its survival outside the host and making it suitable for transmission between hosts [46]. IMV can fuse with the plasma membrane by interacting with glycosaminoglycans on the host cell surface. Phosphatidylserine exposed to the IMV membrane can activate virus macropinocytosis, allowing the virus to enter the host via the endocytosis pathway [40]. Intracellular enveloped viruses (IEVs) are formed when the host cell’s inner membrane envelops the IMV. IEVs via microtubules to move to the cell surface and fuse with the plasma membrane to produce cell‐associated enveloped viruses (CEVs). CEVs can induce actin tails to facilitate their dissemination into neighboring cells or be released from the cell surface to form EEVs for long‐distance dissemination [47]. EEV has a bilamellar structure, and its transfer between host cells is facilitated by an additional but fragile lipid membrane layer on its surface. EEV’s additional surface proteins allow the formation of an entrance fusion complex (EFC), which allows the viral core to enter the cytoplasm of the host cell and to be expelled from the host cell by cytokinesis [46, 48]. The host Golgi plays an important role in MPXV infection: the VPS52 and VPS54 proteins of the Golgi‐associated retrograde protein (GARP) complex are essential for EEV formation [49]; Deficiency of conserved oligomeric Golgi (COG) proteins disrupts the transport of membrane glycosylated proteins, leading to impaired virus‐protein interactions required for viral entry into, or release from the host cell. In particular, COG4 and COG7 are essential for viral fusion [50].

In the context of vertical transmission, a virus’s ability to effectively infect nonproliferating cells is crucial for crossing the placental barrier. For example, CMV has been demonstrated to efficiently infect differentiated, nonproliferating syncytiotrophoblast and cytotrophoblast cells in the placenta, a capability closely linked to its successful placental crossing [51]. Similarly, the Zika virus has also been shown to infect and traverse the placental barrier. For MPXV to achieve vertical transmission, its viral particles (including IMV and EEV) must also possess the ability to infect specific cell types within the placenta. In pregnant women during gestation, Andrieu et al. reported that MPXV infection induced trophoblast cells to form multiple plate‐like or filamentous pseudopods. MPXV particles were present in the interior and at the tips of these pseudopods, which contributed to their specific diffusion into surrounding trophoblast cells via plasma membrane fusion or micropinocytosis [52]. Second, TAM receptors are present in the placenta, and the soluble serum protein Gas6 can mediate the bridging of phosphatidylserine on the viral membrane to the TAM tyrosine kinase receptor on the target cell, causing them to bind and activate the MPXV to enter the placental cells [53]. In addition, it has been demonstrated that placental syncytiotrophoblast cells can induce micropinocytosis by inhibiting mammalian target of rapamycin (mTOR) signaling [38].

The aforementioned invasion mechanisms of MPXV—particularly the “mimicry switch” and the Gas6–TYRO3/AXL/MERTK (TAM) receptor pathway—suggest that detecting soluble Gas6 in maternal serum or TAM receptor expression (e.g., AXL, MERTK) in placental tissue could serve as potential biomarkers for predicting the risk of vertical transmission. Therapeutic interventions targeting this pathway, such as the development of soluble TAM receptor decoys or anti‐Gas6 neutralizing antibodies, represent promising strategies for preventing mother‐to‐child transmission of MPXV. In parallel, pregnant individuals infected with MPXV should be managed as high‐risk pregnancies and receive intensified prenatal monitoring. This includes close surveillance of placental morphology and function via ultrasound and other imaging modalities. The detection of placental structural abnormalities—such as irregular contour, focal thickening, or abnormal echogenicity/calcification—should raise clinical suspicion of possible intrauterine infection and prompt further diagnostic evaluation.

### 5.2. Mechanism of MPXV Replication and Synthesis

Orthopoxviruses have large genomes and replicate in the cytoplasm. They are less dependent on host function than other DNA viruses. However, host cell heat shock factor 1 (HSF1) is phosphorylated during poxvirus infection, translocated to the nucleus, and increases transcription of HSF1 target genes. Activation of HSF1 is supportive for orthopoxvirus replication and is a key host factor for MPXV infection [54].

Upon entry of MPXV into the host cell, its genome is rapidly released by decidualization and replicated in the cytoplasmic endoplasmic reticulum [55]. Proteins directly involved in and essential for viral DNA replication are mostly encoded by MPXV’s genome: the D5 protein is a multistructural domain protein. It consists of a deconjugating enzyme structural domain at its C‐terminal end, and the N‐terminal end contains an archaeal elicitor enzyme (AEP) structural domain that plays a key role in DNA replication [56, 57]. Yaning Li’s team showed that the primase domain in the D5 protein of MPXV plays a key regulatory role in DNA replication, which means that when the primase domain of the D5 protein is disrupted, the dsDNA of MPXV will be unspun into single, thus blocking its DNA replication process [58]. In addition, E5 is a conjugate protein that is closely related to MPXV replication. Weizhen Zhang’s team found that the full‐length MPXV E5 protein forms an asymmetric, self‐repressing hexamer, whose AEP domain prevents the DNA from entering the central channel in the correct orientation, and that truncation of the N‐terminal end restores deconjugate enzyme activity of E5 protein [59]. Given the paramount concern for fetal safety in pregnancy, virus‐encoded proteins that are devoid in human cells—such as the AEP domain of the D5 protein—represent ideal targets for antiviral drug design. Developing small‐molecule inhibitors (e.g., against the viral helicase‐primase) against these targets promises safer therapeutic options against MPXV for both the mother and fetus. By directly inhibiting viral replication and reducing maternal viral load, such strategies could effectively prevent vertical transmission and improve pregnancy outcomes.

### 5.3. Immune Evasion and Immunopathogenesis of MPXV in Pregnancy

#### 5.3.1. Mechanisms of MPXV Immune Evasion

MPXV employs a sophisticated arsenal of viral proteins to disarm both innate and adaptive host immunity, a feature critical to its pathogenesis. In the unique immunological context of pregnancy, these evasion strategies are not only permissive for initial infection but also set the stage for severe placental damage. This section systematically details MPXV’s key mechanisms to suppress interferon (IFN) responses, dampen inflammation, and modulate T‐cell activation, providing the molecular basis for the immunopathology discussed subsequently.

**Figure 4:**
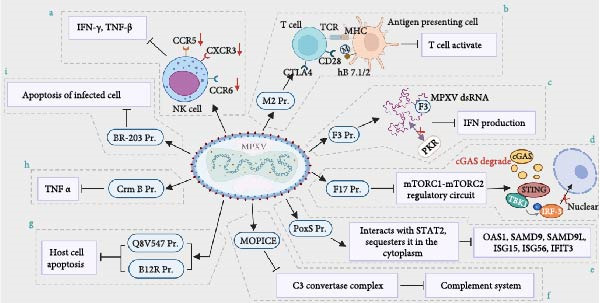
Pathogenic immune evasion mechanisms of MPXV infection. (a) MPXV impairs NK cell function by inhibiting the expression of chemokines (CCR5, CXCR3, CCR6), thereby decreasing IFN‐*γ* and TNF‐*α* secretion. (b) MPXV‐encoded M2 protein can disrupt T cell activation by binding to the hB7.1/2 receptor of APC. (c) The MPXV‐encoded F3 protein blocks the binding of virus‐produced dsRNA and PKR, thereby antagonizing the IFN‐mediated antiviral immune response. (d) MPXV late structural protein F17 inhibits the cGAS‐STING antiviral pathway by dysregulating the mTORC1‐mTORC2 complex. (e) The PoxS fusion protein of MPXV interacts with STAT2 and blocks IFN‐induced expression of antiviral genes. (f) MOPICE encoded by clade I MPXV inhibits complement activation by preventing the formation of C3 convertase. (g) MPXV‐encoded Q8V547 and B12R (OPG046) proteins act as apoptosis regulators and inhibit the apoptosis of host cells. (h) The MPXV‐encoded CrmB (OPG135) protein competitively binds to TNF‐*α*, preventing it from binding with the corresponding receptor, thereby interfering with the extrinsic apoptotic pathway induced by TNF‐*α*. (i) The MPXV‐encoded BR‐203 (OPG015) protein avoids apoptosis in infected lymphocytes and facilitates virus propagation within host cells.

Immune tolerance at the maternal–fetal interface is crucial for the successful progression of pregnancy. Human leukocyte antigen G (HLA‐G) is uniquely expressed in the extrachorionic trophoblast and interacts with multiple receptors on maternal immune cells within the uterus. This interaction is essential for maintaining immune homeostasis at the maternal–fetal interface, thereby preventing detrimental communications that could adversely affect the fetus. Research indicates that embryonic loss during early pregnancy is associated with impaired HLA‐G functionality and inadequate regulation of immune tolerance [60]. Research indicates that trophoblast cells infected with herpes simplex virus (HSV) disrupt antigen presentation through HLA‐G, which subsequently affects immune cell tolerance and damages the maternal–fetal interface, ultimately leading to miscarriages [61, 62]. However, there is a limited body of research investigating the impact of MPXV infection on HLA‐G‐mediated antigen presentation in trophoblast cells. This presents a potential avenue for future studies aimed at elucidating the mechanisms through which MPXV infection may contribute to adverse pregnancy outcomes. Furthermore, understanding the immune evasion strategies employed by MPXV is crucial for the development of antiviral therapies. Current investigations into these immune evasion mechanisms primarily focus on the virus’s ability to inhibit IFN synthesis and antiviral pathways, suppress host inflammatory responses, and modulate apoptotic processes (Figure 4).

MPXV orchestrates a multilayered attack on the type I IFN system, the primary antiviral defense of the host. This suppression is achieved through several viral proteins that target distinct nodes of the IFN signaling cascade. A significant phenomenon during normal pregnancy is the dynamic alteration in cytokine expression and the composition of immune cells at the maternal–fetal interface, which correlates with the progression of gestation. In the early stages of pregnancy, the processes of placental implantation and development lead to an increase in the levels of inflammatory mediators and immune cells within the maternal meconium. By mid‐gestation, the presence of certain anti‐inflammatory effects becomes essential at the maternal–fetal interface, which is linked to modifications in the recruitment of meconium NK cells, M2‐type macrophages, and regulatory T (Treg) cells that play a crucial role in maintaining immune tolerance toward the fetus [63, 64]. Infection with MPXV results in lymphadenopathy, which is characterized by changes in lymphocyte populations, particularly an increase in NK cells. Simultaneously, MPXV infection is linked to a decrease in the secretion of IFN‐*γ*, primarily due to the inhibition of chemokine expression, which compromises the functional capacity of NK cells [65]. Diminished degranulation of NK cells and a reduction in IFN‐*γ* expression during MPXV infection may directly hinder the ability of NK cells to eliminate MPXV‐infected cells [66, 67]. Furthermore, the M2 protein, which is expressed during the early stages of orthopoxvirus infection, reduces the phosphorylation of ERK2 and inhibits the activation of NF‐*κ*B, thereby modulating the NF‐*κ*B‐mediated innate immune response [68]. Yang et al. [29] demonstrated that the M2 protein encoded by MPXV can bind to human B7.1 and B7.2 proteins (hB7.1/2). This interaction disrupts the binding of hB7.1/2 to CD28 and CTLA4, thereby impeding the activation of T‐cells, which is typically facilitated by the co‐stimulatory signals provided by hB7.1/2. This mechanism plays a significant role in the immune evasion strategies employed by MPXV. By this critical co‐stimulatory checkpoint, MPXV effectively paralyzes the primary communication channel required for naïve T‐cell activation and clonal expansion. In the unique immunological context of pregnancy, which is inherently biased toward tolerance to avoid fetal rejection, this targeted disruption of T‐cell co‐stimulation is particularly deleterious. It can catastrophically impair the maternal immune system’s ability to recognize and eliminate MPXV‐infected placental trophoblasts, thereby facilitating unchecked viral replication and dramatically increasing the risk of vertical transmission. The innate immune response is significantly influenced by the type I IFN response. Consequently, various viruses, including the MPXV, evade the antiviral innate immunity of host cells primarily by suppressing the type I IFN response. MPXV expresses the F3 protein, which is homologous to the E3 protein found in CPXV. This protein interacts with double‐stranded RNA (dsRNA), effectively sequestering it from protein kinase R (PKR). This interaction inhibits the activation of PKR, thereby undermining the IFN‐mediated antiviral immune response [69]. In the placenta, where timely IFN responses are critical for limiting viral spread between trophoblasts, this early blockade may allow MPXV to gain an initial foothold. The late structural protein F17 of MPXV disrupts the functioning of the mTORC1 and mTORC2 complexes and impedes the cGAS‐STING pathway, which is responsible for the expression of the interferon‐stimulated gene (ISG) and the subsequent antiviral response [70]. Such broad‐spectrum suppression of ISGs could compromise the placental barrier’s intrinsic resistance to infection. Recent research conducted by Pearl Chan and colleagues has demonstrated that the poxin‐schlafen (PoxS) fusion protein of the MPXV interacts with STAT2, resulting in its sequestration in the cytoplasm. This interaction effectively inhibits the expression of IFN‐induced antiviral genes, including OAS1, SAMD9, SAMD9L, ISG15, ISG56, and IFIT3, thereby allowing the virus to evade the host’s antiviral defense mechanisms [71]. This represents a particularly potent mechanism to evade the localized antiviral defense at the maternal–fetal interface. Collectively, these layered strategies (F3, F17, PoxS) exemplify the profound IFN antagonism that is a hallmark of MPXV pathogenesis, as highlighted in comprehensive reviews on virus–host immunity [72].

Beyond subverting antiviral IFN responses, MPXV actively dampens broader inflammatory and innate immune reactions, which are crucial for alerting the immune system and clearing infected cells. The M2 protein, which is expressed during the early stages of orthopoxvirus infection, reduces the phosphorylation of ERK2 and inhibits the activation of NF‐*κ*B, thereby modulating the innate immune response that is mediated by NF‐*κ*B [68]. In pregnancy, where inflammation must be precisely regulated for placental development, this suppression may paradoxically disrupt immune homeostasis while facilitating viral replication. The complement system plays a crucial role in innate immunity. The monkeypox complement inhibitor (MOPICE), encoded by the COP‐C3L (OPG213) gene of the MPXV clade I, obstructs the assembly of the C3 convertase enzyme complex. This inhibition suppresses the host’s complement system by preventing complement activation, thereby undermining innate immune responses [22]. Together, the suppression of NF‐*κ*B‐driven inflammation and complement activation by M2 and MOPICE undermines two major alarm systems of the host, potentially allowing MPXV to replicate with reduced immunological surveillance within placental tissues.

Moreover, MPXV has the ability to enhance its replication within host cells by modulating the apoptotic processes of these cells. Infected host cells typically limit viral propagation by inducing apoptosis through both intracellular and extracellular pathways. Several mechanisms by which MPXV influences apoptosis have been elucidated. Notably, the Q8V547 protein antigen present in MPXV, along with the serine protease inhibitor‐2 (SPI‐2) homolog encoded by the B12R (OPG046) gene, acts as a regulator of apoptosis, thereby facilitating viral replication in the infected host cells [69, 73]. Secondly, the tumor necrosis factor receptor (TNFR) homolog CrmB (OPG135), encoded by the MPXV, binds competitively to TNF. This interaction may disrupt extrinsic apoptotic pathways, as CrmB (OPG135) lacks the requisite signaling structure [74]. Finally, the identification of BR‐203 (OPG015), a homolog of the Mucovirus M‐T4 gene found in MPXV, suggests that it encodes a protein potentially involved in the evasion of apoptosis in infected lymphocytes, thereby facilitating the dissemination of the virus within host cells [19]. The viral strategy to suppress apoptosis is critical for establishing sustained infection. In an obstetric context, this mechanism suggests that upon infecting placental trophoblasts, MPXV can persist and replicate, thereby compromising placental function. Such dysfunction may manifest clinically as fetal growth restriction, oligohydramnios, or even fetal demise. Consequently, the management of MPXV‐infected pregnant individuals should encompass not only antiviral strategies but also vigilant monitoring of fetal well‐being. Theoretically, inhibitors targeting viral anti‐apoptotic proteins like SPI‐2 could restore programed cell death in infected cells and curb replication; however, their application in pregnancy mandates extremely rigorous safety evaluation.

The potent arsenal of MPXV immune evasion mechanisms explains the frequent failure of the maternal immune system to clear the infection, particularly within the adapted immunological landscape of pregnancy. This reality dictates a two‐pronged clinical approach: prioritizing pre‐emptive vaccination for women of childbearing age, and providing infected pregnant patients with timely passive immunotherapy to bolster their transiently compromised antiviral immunity. Concurrently, tracking peripheral immune markers like NK cell function and IFN‐*γ* levels can offer critical insights into disease severity and treatment response.

Beyond the pregnancy‐specific mechanisms discussed herein, MPXV pathogenesis must be viewed within the broader host–virus interplay. As Dwivedi et al. [75] highlight, the virus’s subversion of innate and adaptive immunity presents universal challenges in diagnostics and treatment. These insights complement our discussion, emphasizing that advancements in understanding these shared mechanisms—such as immune evasion and T‐cell exhaustion—will inform the development of diagnostics and therapeutics beneficial for all vulnerable groups, including pregnant women.

#### 5.3.2. From Immune Evasion to Placental Damage in MPXV Infection

While MPXV employs immune evasion mechanisms—notably suppressing IFN responses and dysregulating inflammatory pathways—to establish infection, this ultimately precipitates a dysregulated immunopathogenesis at the maternal–fetal interface. This aberrant response is the direct cause of placental damage and fetal compromise.

First, the potent pro‐inflammatory factors released by MPXV infection—such as TNF‐*α*, IL‐6, and IL‐1*β*—directly cause placental damage. Among these, TNF‐*α* directly induces apoptosis in syncytiotrophoblast cells [76]. This disruption of placental barrier integrity not only facilitates viral entry into the fetal circulation but also compromises the placenta’s core function of maintaining nutrient and gas exchange, ultimately resulting in fetal growth restriction and fetal death. This inflammatory milieu further exacerbates placental damage by recruiting neutrophils and inflammatory monocytes/macrophages to the decidua [77], which release proteolytic enzymes and reactive oxygen species, compounding the cytokine‐initiated destruction [77, 78].

MPXV infection disrupts key immune cells at the maternal–fetal interface, driving adverse pregnancy outcomes. It impairs the vascular remodeling function of decidual NK cells, which can lead to inadequate placental perfusion and fetal ischemia [65, 79]. Simultaneously, the virus‐induced proinflammatory environment suppresses Treg cell activity [29], potentially unleashing harmful effector T‐cell responses against placental or fetal antigens. Together, these disruptions compromise immune tolerance and vascular support, contributing to miscarriage and stillbirth.

MPXV infection activates the complement cascade, leading to deposition of membrane attack complexes (MACs) on trophoblast cell surfaces and triggering direct cell lysis. Concurrently, generated anaphylatoxins (such as C5a) exacerbate local inflammation by recruiting and activating immune cells [80]. This complement‐mediated injury mechanism likely explains the punctate hemorrhages and villous destruction observed in the placentas of infected pregnant women.

## 6. Summary and Prospect

Comparative clinical data indicate that pregnancy significantly exacerbates the risks associated with MPXV infection, distinguishing it from the typically self‐limiting disease course observed in nonpregnant individuals. Future prospective cohort studies involving pregnant women are needed to systematically quantify this disparity in risk. The implications of MPXV infection for pregnant women and their pregnancy outcomes have become a significant concern in the field of global public health. Research and documented cases have confirmed the potential effects of MPXV on both the fetus and neonate. Specifically, maternal infection with MPXV during gestation is associated with an increased risk of adverse pregnancy outcomes, including spontaneous abortion, stillbirth, and congenital infections. While there has been some investigation into maternal infections caused by the MPXV, numerous significant questions remain unresolved. The recent monkeypox outbreak was predominantly attributed to clade II; however, despite clade IIb being considerably less pathogenic than clade I, existing studies and case reports indicate its potential association with adverse pregnancy outcomes. There is a scarcity of clinical data pertaining to clade IIb, necessitating further research involving cohorts of pregnant women, as well as an examination of placental pathology and viral load, to ascertain whether vertical transmission from mother to fetus occurs and whether it contributes to negative pregnancy outcomes. Additionally, the mechanisms by which MPXV targets the placenta warrant further investigation. For instance, the role of the TAM receptor in MPXV’s placental infection requires additional experimental validation. Furthermore, the potential of regulatory molecules associated with trophoblast giant cells, such as mTOR signaling, as therapeutic targets remains to be elucidated. Although the replication‐associated proteins of MPXV, specifically E5 and D5, have been studied in cellular contexts, there is limited understanding of the molecular mechanisms underlying the precise assembly and operational stages of the complete replicative machinery.

Current research on the mechanistic aspects of the MPXV suggests the potential for developing targeted interventions. Specifically, antiviral therapies that utilize small‐molecule drugs targeting critical viral replication proteins, such as the D5 primase structural domain or the host factor HSF1, may be effective in treating MPXV infections. However, it is essential to evaluate the safety of these treatments in pregnant women. Furthermore, the immunogenicity of existing Modified Vaccinia Ankara (MVA) vaccines in pregnant populations, as well as their efficacy in preventing vertical transmission, remains unclear and requires thorough investigation through clinical trials involving pregnant individuals. Concurrently, benefits and risks must be carefully weighed based on placental injury mechanisms, with shared decision‐making involving the patient. For the clinical management of pregnant women infected with MPXV, we propose the following recommendations: Enhanced fetal monitoring (such as serial growth ultrasounds and placental assessments) should be implemented for infected pregnant women. Risk stratification based on disease severity, gestational age at infection, and viral load should be conducted to identify high‐risk pregnancies requiring multidisciplinary management.

The infection of pregnant women with MPXV poses a dual challenge, marked by both clinical urgency and scientific complexity. Understanding the pathogenic differences between clades I and IIb, the mechanisms of placental invasion specific to the virus, and the strategies employed for immune evasion will provide a foundation for early detection and targeted intervention in pregnancy‐related complications. Moving forward, it is essential to promote multicenter collaborations and utilize advanced technologies to clarify the molecular interactions between MPXV, the placenta, and the fetus, with the ultimate goal of improving health outcomes for both mothers and infants. Foremost among these priorities is addressing the pronounced evidence gap for clade IIb. This necessitates an immediate global commitment to establishing multicentric pregnancy registries and prospective cohort studies to definitively quantify vertical transmission risk and adverse outcomes.

Significant barriers impede the global implementation of MPXV interventions, particularly in under‐resourced regions disproportionately affected by the disease. Key challenges include the cold‐chain requirements of MVA vaccines in areas with unstable infrastructure, the cost and complexity of novel antivirals in poorly equipped clinics, and overarching issues of poverty and weak health systems. Moving forward, priority must be given to developing heat‐stable vaccines, affordable oral drugs, and integrated service delivery models to ensure equitable MPXV control through reinforced primary healthcare.

## Conflicts of Interest

The authors declare no conflicts of interest.

## Author Contributions

Qingliang Zheng and Jingyi Wu conceived this review. Jingyi Wu and Fangbin Huang wrote the manuscript. Jingyi Wu made the figures and tables.

## Funding

This study was supported by the National Natural Science Foundation of China (82371677), Natural Science Foundation of Guangdong (2024A1515013144), Shenzhen Science and Technology Program (JCYJ20220530144208019, JCYJ20230807111301004, and JCYJ20240813150622030), and Futian Healthcare Research Project (No. FTWS2022005 and FTWS012).

## Data Availability

Data sharing is not applicable to this article as no datasets were generated or analyzed during the current study.
